# Validation of an RPHPTLC-Densitometric Method Using Silica Gel 60 RP18WF_254_ for Simultaneous Determination of Nicotinamide in Selected Pharmaceutical Formulations

**DOI:** 10.1155/2015/631025

**Published:** 2015-03-05

**Authors:** Małgorzata Dołowy, Alina Pyka

**Affiliations:** Institute of Analytical Chemistry, Department of General and Analytical Chemistry, School of Pharmacy and the Division of Laboratory Medicine, Medical University of Silesia in Katowice, 4 Jagiellońska Street, 41-200 Sosnowiec, Poland

## Abstract

This research study describes the applicability of silica gel 60 RPW18F_254_ plates for the development and validation of new, simple, economic, accurate, and precise RPHPTLC-densitometric method suitable for the quantification of nicotinamide (as *Vitamin PP*) in three marketed preparations. The mobile phase used was methanol-water in volume composition 3 : 7. Detection wavelength was 200 nm. The proposed method was validated according to ICH guidelines and also based on Ferenczi-Fodor and Konieczka reports. Results were found to be linear over a range of 1.00 to 2.00 *μ*g/spot. Limit of detection (LOD) and limit of quantification (LOQ) were 0.15 *μ*g/spot and 0.45 *μ*g/spot, respectively. The percent content of nicotinamide in the investigated preparations was found to be 99.2% (*Product 1*), 99.3% (*Product 2*), and 99.4% (*Product 3*). Developed method is accurate and precise (CV < 3%) and may be successfully applied for the quality control of pharmaceutical formulations containing nicotinamide in the presence of its derivatives, such as N,N-diethylnicotinamide, N-methylnicotinamide, and nicotinic acid.

## 1. Introduction

Numerous research and review papers show that among various sorbents, such as cellulose, chitin, aluminum, polyamide, and polymers, silica gel is by far the most frequently used stationary phase in different separation techniques including liquid chromatography. Silica gel used in liquid chromatography is porous, synthesized material [1]. The presence of silanol groups at the surface of silica gel which play a role of adsorption-active centers causes that this sorbent is suitable as stationary phase in different chromatography in normal phase, reversed phase, ion chromatography, or hydrophilic interaction liquid chromatography. In addition to this, ability of mentioned silanol groups to react with various reagents is widely applied to surface modification of silica gel [1–4]. A number of papers prepared by Bocian and coworkers confirmed that the columns packed with chemically modified silica gel, such as cholesterol-bonded, alkyl-phosphate bonded, ester-bonded silica gel, and others are suitable for liquid chromatographic separation of various biologically active compounds [5–10]. Moreover, different types of silica gel are used as the dominant stationary phases in TLC and HPTLC techniques [1, 4]. Today, the commercially available TLC and HPTLC plates are made with a thin-layer of silica gel unmodified or modified, for example, by attaching to silica gel surface either nonpolar alkyl chain (e.g., C4, C8, C18, and C30) or a nonpolar groups, including –NH_2_, –CN, and –Diol. Thin-layer of silica gel can also contain fluorescent indicator F_254_. In recent years, there have been wide studies for the TLC-densitometric analysis of different biological significance compounds. Among number of adsorbents, silica gel plates and its derivatives (connection with –NH_2_, –CN, –Diol or with RP18, RP8, and RP2, resp.) are an efficient adsorbent for normal (NP) and also reversed-phase (RP) thin-layer chromatographic analysis of drugs [1, 4].

Therefore, the main goal of present work was to confirm the applicability of an RPHPTLC-densitometric method using commercially available silica gel 60 RP18WF_254_ plates to determine the content of nicotinamide in its selected pharmaceutical formulations in tablets and also as injection dosage forms.

Nicotinamide (chemically known as pyridine-3-carboxamide) is a solid (white powder) freely soluble in water (about 691–1000 g/L at 20°C) with pKa = 3.35 (Dissociation Constant at 20°C) and vapour pressure 31.4 hPa (at 25°C). It has melting point 127–130°C and boiling point 157°C (0.066 Pa) [11, 12]. The lipophilicity (logP) of this compound (a physicochemical property important for correlation of organic molecule activity with its biological behavior) measured by use of traditional shake-flask method in n-octanol-water system is −0.37. Theoretical partition coefficients calculated by means of various software products are as follows: −0.29 (MlogP), −0.32 (AlogP), −0.34 (XlogP2) = −0.37 (XlogP3), −0.45 (AlogPs), and −0.48 (miLogP) [12].

It is well known that nicotinic acid and its derivatives, for example, nicotinamide, belong to the water soluble vitamin B family. Both compounds indicate the activity of vitamin B_3_ which is commercially available in various pharmaceutical formulations and also diet supplements. Described nicotinamide represents an important therapeutic option for the treatment of central nervous system disorders and synthesis of some steroid hormones. In Polish markets, nicotinamide is available as* Vitamin PP* in tablets and injections for different use (e.g., intramuscular, intravenous, or under the skin).

Several nicotinamide determination procedures for biological samples (e.g., urine or human serum), food products, cosmetic products, and also pharmaceutical formulations have been reported. These include methods: UV-VIS spectrophotometry, gas chromatography, microemulsion electrokinetic capillary chromatography, and high-performance liquid chromatography combined with different detection modes and various types of silica gel as stationary phases [13–38]. According to our knowledge until today, there are only two reports which have been published in 1985 and in 1986 describing the quantification of nicotinamide by densitometric thin-layer chromatography in vitamin preparations [39, 40].

This research aimed to develop and validate an RPHPTLC-densitometric method with the use of silica gel RP18WF_254_ plates for simultaneous determination of nicotinamide in selected pharmaceutical dosage forms. Full method validation was presented in terms of specificity, selectivity, detection limit, quantification limit, linearity, accuracy, and precision according to latest ICH guidelines (International Conference on Harmonization) and also others, for example, Ferenczi-Fodor and Konieczka et al. reports [41–44]. It would be advantageous in the routine laboratory control of nicotinamide tablets and also its injection preparations.

## 2. Materials and Methods

### 2.1. Standards and Chemicals

Reference standards of investigated nicotinamide (NAM) and also its derivatives, such as N-methylnicotinamide (MNAM), N,N-diethylnicotinamide (DENAM), and nicotinic acid (NA), were procured from Sigma-Aldrich (St. Louis, MO, USA). Ethanol absolute (≥99.8%) and methanol for HPLC were obtained from POCh (Gliwice, Poland). Distilled water was produced by Institute of Analytical Chemistry, School of Pharmacy, and the Division of Medicine Laboratory (Sosnowiec, Poland). All chemicals and reagents were analytical grade.

### 2.2. Pharmaceutical Formulations

Tablet preparations containing nicotinamide (as* Vitamin PP*) in quantity 200 mg/tablet manufactured by two producers (*Product 1* and* Product 2*) and also nicotinamide in quantity 100 mg/ampoule as injection dosage form (*Product 3*) were used in this study.

### 2.3. Materials

RP-HPTLC plates, 10 cm × 10 cm glass plates, are precoated with 0.20 mm layers of silica gel 60 RP18WF_254_ (E. Merck, Darmstadt, Germany, Art. 1.13124).

### 2.4. Apparatus

The used equipment is as follows:

- Densitometer TLC Scanner 3 with WinCATS 1.4.2 software, manufacturer: Camag (Muttenz, Switzerland),
- IKA Ultra-Turrax Tube Drive Workstation with BMT-20-S Tube for grinding with balls of stainless steel,
- TLC chamber: twin-trough chamber for 20 cm × 10 cm plates (Art. 0.222.5221, Camag, Muttenz, Switzerland),
- the 5 *μ*L Camag micropipettes (Muttenz, Switzerland) used to apply the solutions to the plates.

### 2.5. Preparation of Standard Solutions and Samples for RPHPTLC Analysis

Ten tablets of appropriate pharmaceutical preparation (*Product 1* or* Product 2*) were weighed and next placed into separate container (grinder with four balls) in order to grind the tablets down by means of Ika Ultra Turrax Tube Drive apparatus. All tablets were ground for 45 minutes with a speed equal to 4000 rpm. After this time, the powdered tablets containing 200 mg of nicotinamide were extracted using 10 mL of ethanol absolute (99.8%) for 29 minutes in the same apparatus with the speed 4000 rpm. Next, the obtained extracts from* Product 1 *and* Product 2* were filtered through a medium-density filter (Whatman) to volume flasks (50 mL) and replenished with the use of ethanol absolute to demanded volume. Sample solution of* Product 3* (injection) was prepared by dissolving one ampoule containing 100 mg of nicotinamide in ethanol absolute in volume flask to volume 25 mL. These solutions were used in further study for the preparations of different dilutions of studied nicotinamide at concentrations, 1.75 mg/5 mL, 1.50 mg/5 mL, and 1.25 mg/5 mL, which have been spotted in quantity 5 *μ*L onto chromatographic plates.

### 2.6. RPHPTLC-Densitometric Quantification of Nicotinamide in Pharmaceutical Formulations

Reference standard solution of investigated nicotinamide and also its sample solutions coming from marketed products were spotted in quantity 5 *μ*L by means of micropipettes onto chromatographic plates precoated with 0.20 mm thin layer of silica gel 60 RP18WF_254_ (E. Merck, Art. 1.13124). RPHPTLC plates were then developed with mobile phase consisting of methanol-water (3 : 7, v/v) to the distance 75 mm. Linear ascending development was used in TLC twin-trough glass chamber for 20 cm × 10 cm plates (Camag) saturated with the solvent system about 30 minutes at room temperature 20 ± 2°C before use. After development, the plates were dried in fume cupboard for 24 hours. Both, densitometric and spectrodensitometric analyses were performed using a Camag TLC Scanner 3 (Muttenz, Switzerland) which was controlled by WinCATS 1.4.2 software. All spectrodensitometric measurements were conducted in reflectance absorbance mode in the wavelength range of 200 nm to 400 nm. Densitometric scanning of nicotinamide was conducted at 200 nm. The source of radiation was deuterium lamp. The scanning speed was 20 nm/s and the data resolution was 1 nm/step. The slit dimension was kept at 8.00 mm × 0.30 mm. Each analysis was repeated three times.

### 2.7. Method Validation

The developed RPHPTLC-densitometric method was validated as per the International Conference on Harmonization guidelines (ICH) Q2 (R1) and also in accordance with Ferenczi-Fodor and Konieczka reports for specificity, linearity, range, LOD, LOQ, precision, and accuracy [41–44]. The robustness study was not performed in this work because our preliminary study showed that in the case of applied reversed-phase system a small variation in chromatographic conditions such as the change of amount of mobile phase used (±5%) or methanol content (±0.1 mL) and others (e.g., type of chromatographic chamber, time of development, and time from development to scanning) did not influence significantly on obtained results.

#### 2.7.1. Specificity and Selectivity

The specificity and selectivity of the method were determined by developing appropriate chromatographic conditions (e.g., kind of chromatographic plates for RPHPTLC and mobile phase composition) which enabled satisfactory separation of nicotinamide (NAM) from its related substances: N,N-diethylnicotinamide (DENAM), N-methylnicotinamide (MNAM), and nicotinic acid (NA). In order to estimate the applicability of proposed chromatographic conditions for the complete separation of NAM from its above-mentioned derivatives (DENAM, MNAM, and NA), the results of densitometric analysis were used to calculate the separation factor (*R*_*S*_)—see 1 for each pair of examined compounds: DENAM/MNAM, MNAM/NAM, and also NAM/NA:

(1) $$\begin{array}{c} R_{S}=\frac{2d}{w_{b1}+w_{b2}}, \end{array}$$

where *d* is the distance between the centers of two adjacent chromatographic bands and *w*_*b*1_ and *w*_*b*2_ are the bandwidth at base.

#### 2.7.2. Linearity and Range

Linearity was evaluated by applying eight stock solutions of examined nicotinamide on the silica gel 60 RPHPTLC plates (RP18WF_254_) in quantity 5 *μ*L. The concentration of applied solutions was placed in the range from 0.16 mg/mL to 0.44 mg/mL. Chromatographic plates were developed by means of methanol-water in volume composition: 3 : 7 as mobile phase. Each analysis was repeated six times. Standard calibration plot was constructed by plotting peak area ratio *A* [AU], measured for each concentration of analyte versus amount of nicotinamide in *μ*g/spot.

#### 2.7.3. Accuracy

This parameter was evaluated by measurement of recovery. Accurately known amount of reference standard of nicotinamide in quantity, 80%, 100%, and 120%, was added to the powdered tablets and also into injection solution containing proper amount of nicotinamide. Next, both extracts from tablets and injection solution were chromatographically examined under optimized conditions. Six different analyses (*n* = 6) were performed in this step. Accuracy of developed method was expressed as recovery (*R*) given in [%] and as coefficient of variation (CV, %).

#### 2.7.4. Precision

Intraday precisionof the method was verified by analysis of three replicates of three sample solutions (extracts from tablets and from injection solution) at concentration: 0.25 mg/mL, 0.30 mg/mL, and 0.35 mg/mL in a short time (during the same day). Intermediate (interday) precision was described with the use of three drug sample solutions at the same concentration which are presented above. The measurements were done for these sample solutions during two weeks. In each case, 5 *μ*L of respective solution was applied. All analyses were performed three times. On the basis of obtained peak areas, the precision of developed method was evaluated as the relative standard deviation (coefficient of variation, CV (%)).

#### 2.7.5. Limit of Detection (LOD) and Limit of Quantification (LOQ)

Limit of detection of examined nicotinamide was determined by applying specific calibration curve which has been prepared on the basis of stock solutions at concentration placed in the detection range. The following reference standards were used: 0.04 mg/mL, 0.06 mg/mL, and 0.08 mg/mL. 5 *μ*L of each solution was spotted on chromatographic plates corresponding to 0.20, 0.30, and 0.40 *μ*g of nicotinamide/spot. The results are mean values of three measurements.

LOD and LOQ were calculated as

(2) $$\begin{array}{c} \text{LOD}=\frac{3.3\times \sigma }{S}\\ \text{LOQ}=\frac{10\times \sigma }{S}, \end{array}$$

where *S* is the slope of the calibration curve and *σ* is the standard deviation of the intercept of specific calibration curve (*S*_*a*_).

Statistical evaluations of the obtained results (e.g., calibration plots) were analyzed by Statistica v 10.0 PL (StatSoft, Kraków, Poland).

## 3. Results and Discussion

Nicotinic acid amide is an organic compound which plays important role in health. It is broadly available in pharmacies as* Vitamin PP* in the form of tablets and also as injection solution. Therefore, there is a need to develop a simple in use and not expensive analytical method, for example, thin-layer chromatography in reversed-phase system combined with densitometry enabling the quantitative determination of nicotinamide in tablets and in injection formulations. In the present paper, an attempt has been made to develop and validate a new, simple, cost-effective, and accurate RPHPTLC-densitometric method for accurate determination of nicotinamide (*Vitamin PP*) in both marketed formulations: tablets and injection solution. This work is continuation of previous extensive study by Pyka et al. [45–50] concerning the use of TLC-densitometry in evaluation of chemical stability and also for the separation of various nicotinic acid derivatives. Numerous papers prepared by Pyka and coworkers affirmed that different mobile and also stationary phases including RP18WF_254_ plates and TLC densitometry are suitable for the separation and to examine the chemical stability of these compounds [45–50].

The proposed RPHPTLC-densitometric method with the use of silica gel 60 RP18WF_254_ as stationary phase and mixture of methanol-water (3 : 7, v/v) for the quantification of nicotinamide (namely,* Vitamin PP*) in selected pharmaceutical formulations (*Product 1*,* Product 2*, and* Product 3*) was validated as per the ICH guidelines and also based on Ferenczi-Fodor and Konieczka reports [41–44].

### 3.1. Selectivity and Specificity

**Figure 1:**
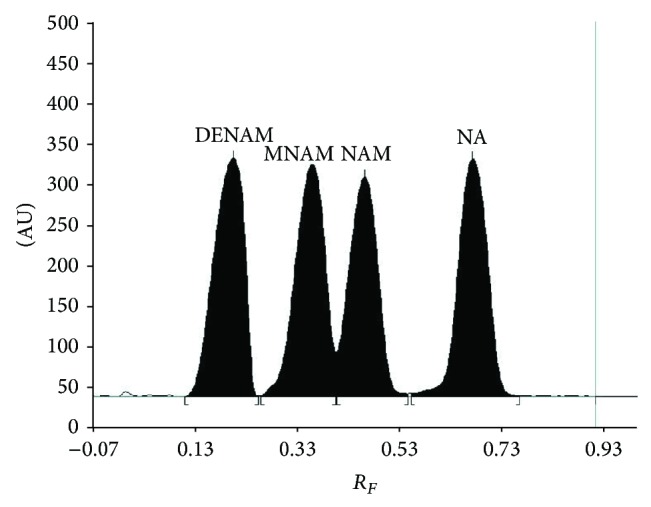
Densitogram of RPHPTLC separation of reference standard of examined nicotinamide (NAM) from its related substances: N,N-diethylnicotinamide (DENAM), N-methylnicotinamide (MNAM), and nicotinic acid (NA) obtained on silica gel 60 RP18WF_254_ and with the use of methanol-water (3 : 7, v/v) as mobile phase.

**Figure 2:**
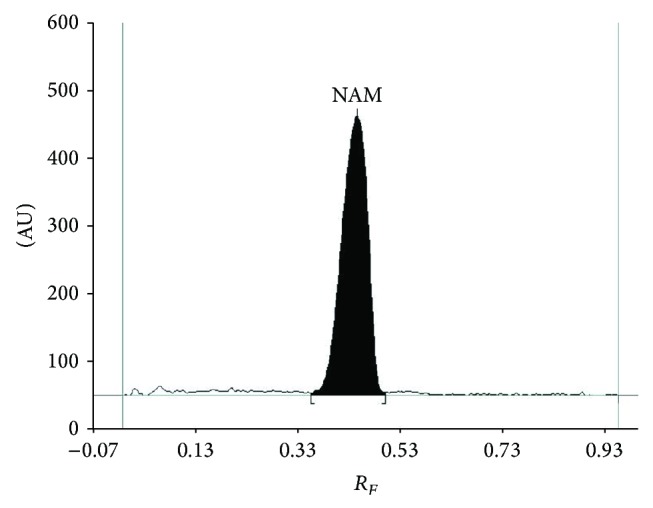
Densitogram of nicotinamide coming from commercially available injection solution (*Vitamin PP*) obtained on silica gel 60 RP18WF_254_ and with the use of methanol-water (3 : 7, v/v) as mobile phase and recorded at 200 nm.

**Figure 3:**
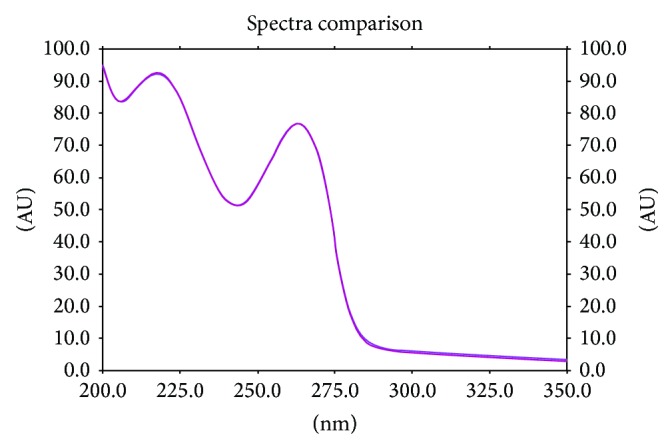
Comparison of the spectrodensitograms of reference standard of nicotinamide and its injection dosage form (*Vitamin PP*) obtained using developed RPHPTLC-densitometric method.

An effort has been made to develop a mobile phase which allowed to obtain complete separation of examined nicotinamide (NAM) from its related substances, such as N,N-diethylnicotinamide (DENAM), N-methylnicotinamide (MNAM), and nicotinic acid (NA) which could be presented in nicotinamide pharmaceutical preparations as potential impurities or its degradation products. Based on previous investigation by Pyka and Klimczok [45], among different mobile phases used, the best results of separation of four discussed compounds, NAM, DENAM, MNAM, and NA, have been achieved on silica gel 60 RP18WF_254_ and methanol-water in volume composition 3 : 7 as mobile phase. This mobile phase enabled obtaining the desired *R*_*F*_ value for examined compounds: *R*_*F*(NAM)_ = 0.43 ± 0.01, *R*_*F*(DENAM)_ = 0.18 ± 0.01, *R*_*F*(MNAM)_ = 0.33 ± 0.01, and *R*_*F*(NA)_ = 0.68 ± 0.01. Although obtaining well-separated peaks of four studied substances using this solvent system confirmed the separation factor (*R*_*S*_ ≥ 1.00) which has been calculated for each pair of examined compounds, the Rs value for the pairs of examined compounds DENAM/MNAM, MNAM/NAM, and NAM/NA was found to be *R*_*S*(DENAM/MNAM)_ = 1.52, *R*_*S*(MNAM/NAM)_ = 1.00 and *R*_*S*(NAM/NA)_ = 2.00, respectively, Figure 1. The wavelength used for detection of nicotinamide was 200 nm. Presented data of *R*_*S*_ demonstrate the specificity and also selectivity of proposed method. Therefore, the described chromatographic conditions were applied in further study to determine nicotinamide (NAM) as* Vitamin PP* in tablets (*Product 1 *and* Product 2*) and in injection formulation (*Product 3*). Densitograms obtained for nicotinamide coming from extract of injection solution (in Figure 2) and also those obtained from tablets indicates that applied reversed-phase TLC combined with densitometry (at *λ* = 200 nm) on silica gel 60 RP18WF_254_ with the use of methanol-water (3 : 7, v/v) is selective and specific. On the basis of Figure 2 which represents the results of nicotinamide analysis in injection solution, it could be observed that there is no interfering peak coming from inactive ingredients and also from nicotinamide derivatives (e.g., N,N-diethylnicotinamide, N-methylnicotinamide, and nicotinic acid) in analyzed preparations. No significant interference observed for nicotinamide in three examined samples and in blank sample confirmed selectivity of proposed method. The mean value of *R*_*F*_ determined for NAM coming from tablets and from injection solution is in agreement with that obtained for its reference standard (*R*_*F*_ = 0.43). Thus, it could be concluded that obtained results of peak areas (AU) of investigated nicotinamide are reliable and may be applied in further quantitative determination of this substance in its formulations (e.g., tablets, injection). Moreover, our spectrodensitometric study of reference standard of nicotinamide and its pharmaceutical preparations indicates that comparison of spectrodensitograms recorded at optimum wavelength for nicotinamide (equal to 200 nm) is suitable for determining identity of examined nicotinamide.  Figure 3 demonstrates well compatibility of spectrodensitogram coming from reference standard of NAM and its extract from injection formulation. Similar agreement of both spectra for standard and sample was obtained for tablets. In all cases the correlation coefficient between both spectra which has been calculated using WinCats 1.4.2 program was ≥0.999.

### 3.2. Linearity and Range

**Figure 4:**
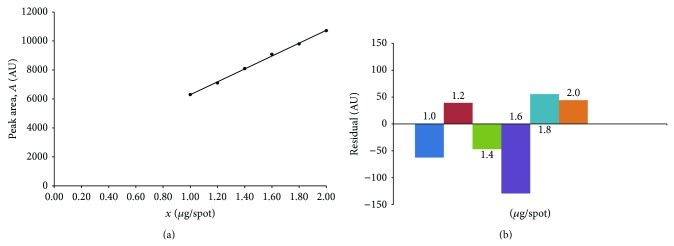
Calibration plot (a) and plot of residuals (b) for nicotinamide in the linear working range.

Linearity relationship was observed by plotting peak areas (AU) recorded densitometrically at 200 nm versus the accurately known amount of nicotinamide in *μ*g/spot. The results were found to be linear in the range of 1.00 ÷ 2.00 *μ*g of nicotinamide/spot (Figure 4(a)). The plot of residuals versus the amount of nicotinamide presented in Figure 4(b) showed that obtained residuals are placed above and below zero line of residuals. Thus, it confirmed good linear relationship with equation:

(3) $$\begin{array}{c} A=4447.3\left(\pm 92.9\right)\cdot x+1844.6\left(\pm 142.9\right), \end{array}$$

where *A* is the peak area ratio of nicotinamide [AU], *x* is the amount of nicotinamide [*μ*g/spot] and mean *r* (correlation coefficient) is better than 0.99 (*r* = 0.9991), for *n* = 6, *F* = 2291, *s* = 77.7, and *P* < 0.0001—Figure 4(a).

### 3.3. Accuracy

Because the composition of all formulation excipients in examined nicotinamide drugs is not well known, accuracy of the method was carried out by standard addition method. The experiment was conducted in triplicate at three different concentrations of nicotinamide: 80%, 100%, and 120% which was added to each studied sample. For each commercial preparation independent testes in triplicate were carried out. When well-known amount of nicotinamide was spiked to the sample of* Product 1* (e.g., 80%, 100%, and 120%), the determined amount of nicotinamide was found from 96.1% to 99.1%, from 95.9% to 99.3%, and from 95.6 to 99.1%, respectively. Thus, mean value of recovery in each case was 98.0%, 97.6%, and 97.5% with the CV (coefficient of variation) less than 1.5% (1.16%, 1.40%, and 1.37%).

The observed results obtained for* Product 2* (after addition of 80%, 100%, and 120% of analyte) were found in the range from 96.2% to 101.3%, from 96.8% to 99.2%, and from 96.9% to 100.9%, respectively. Calculated recoveries were 98.7%, 98.0%, and 98.7% with the CV [in %] less than 2% (1.99%, 0.89%, and 1.42%).

In the case of* Product 3*, the obtained results (in the presence of spiked 80%, 100%, and 120% of analyte) existed in the range from 96.1% to 99.1%, from 95.9% to 99.3%, and from 95.6 to 99.1%. Average values of calculated recoveries were changed from 98.0% by 97.6% to 97.5% with the CV [in %] less than 1.5% (1.16%, 1.40%, and 1.37).

To summarize the results of accuracy of described method which is expressed as recovery and also as coefficient of variation in percent [CV] at the 95% confidence level, it could be concluded that the developed RPHPTLC-densitometric method is accurate and reproducible. The results of the validation are presented in Table 1.

### 3.4. Precision (Repeatability)

The precision of the method was calculated in terms [%] relative standard deviation (coefficient of variation, CV) of intraday and interday precision of the method. Intraday and interday precision of the assay were determined using three samples replicates of nicotinamide preparations (*Product 1, Product 2*, and* Product 3*) at different concentrations of nicotinamide (0.25, 0.30, and 0.35 mg/mL). Precision was estimated by densitometric measurements of the peak areas obtained for analyzed samples and it was expressed as coefficient of variation (CV, %). The validation data showed that coefficient of variation for the determination of peak areas in the three examined pharmaceutical formulations was placed in the following range:

- from 1.02% to 1.52% (intraday precision) and from 1.75% to 2.42% (interday precision) for* Product 1* (tablets),
- 0.78% do 1.22% (intraday precision) and from 1.32% to 1.78% (interday precision) for* Product 2* (tablets),
- from 0.89% to 1.33% (intraday precision) and from 0.98% to 1.76% (interday precision) for* Product 3.*

The results of CV less than 3% confirmed precision of proposed method.

### 3.5. Sensitivity (Limit of Detection (LOD) and Limit of Quantification (LOQ))

The LODs and LOQs of nicotinamide were calculated on the basis of standard deviation of the intercept of specific calibration curve according to the formulae 2. The limit of detection and the limit of quantification determined by the use of developed method were found to be 0.15 *μ*g/spot and 0.45 *μ*g/spot, respectively. Because the standard deviation depends on concentration (amount) of analyzed compound, there is a need to check the correctness of obtained LOD values with the use of the following relationships observed between LOD value and concentration of examined substance [44]:

(4) $$\begin{array}{c} 10\times \text{LOD}>\text{C}\\ \text{LOD}<C, \end{array}$$

where LOD is limit of detection and *C* is concentration of analyzed substance in the reference samples.

Our results of LOD fulfilled the conditions presented in 4. Thus it confirms that the amount of nicotinamide used in preparation of specific calibration plot was well selected.

### 3.6. Quantitative Determination of Nicotinamide in Commercial Samples (as* Vitamin PP*)

The validity of the proposed RPHPTLC method in combination with densitometry was applied for the accurate determination of nicotinamide in its pharmaceutical preparations:* Product 1* and* Product 2* (tablets) and* Product 3* (injection solution). In this study, sample solutions of the investigated preparations in quantity of 1.50 *μ*g of nicotinamide/spot were chromatographed on silica gel 60 RP18WF_254_ and with the use of methanol-water (3 : 7, v/v). The results of peak areas which have been densitometrically recorded for each sample at 200 nm were used to calculate the nicotinamide content using the equation of prepared regression plot 3. Statistical analysis of obtained results in the three marketed preparations in *μ*g/spot or in mg/tablet (mg/ampoule), respectively, was listed in Table 1.

The data presented in Table 1 demonstrate that generally the content of nicotinamide (*Vitamin PP*) in respective commercial preparations in relation to that declared by manufacturers was found to be 99.2% (*Product 1*), 99.3% (*Product 2*), and 99.4% for* Product 3*. Hence, it realizes the criteria required by Pharmacopoeia for nicotinamide content which should be placed in the range from 95% to 105% [51]. Because the elaborated method shows characteristic well with acceptable limits, it may be successfully adopted in routine quality control of nicotinamide formulations in form of tablets and also as injection solution.

As was described in introduction part of this work, several methods including high-performance liquid chromatography (HPLC) and also gas chromatography (GC) were described for the analysis of nicotinamide and its derivatives in biological samples, food products, and also in selected pharmaceutical formulations. Based on the analysis of data presented in this work and those obtained by use of other chromatographic techniques (e.g., HPLC and GC), it could be observed that the limit of quantification for nicotinamide by use of HPLC-UV was found to be in *μ*g/mL (e.g., from 11 to 34 *μ*g/mL) [32]. A gas chromatographic method enabled determination of nicotinamide content in the range from mg/mL to *μ*g/mL [33]. Compared of these results with that which have been obtained by use of proposed RPHPTLC-densitometric method, it could be suggested that HPLC and GC show better sensitivity for the quantification of nicotinamide in pharmaceutical formulations and also in diet supplements, but they are more expensive and time-consuming. The limit of quantification by proposed RPHPTLC-densitometry method is higher, but it is enough for the purpose of pharmaceutical analysis of preparations containing usually from 100 to 200 mg of nicotinamide in tablet/ampoule. The main advantage of newly developed RPHPTLC-densitometric method in relation to HPLC and GC methods and also to earlier reported nicotinamide study by TLC (in 1985-1986) is its simplicity (one step sample preparation is needed), low cost and additionally the quantification is performed in UV, without derivatization (visualizing reagent is not necessary) [39, 40]. Although, the validation process confirmed that the method is specificity, selectivity and allows quantifying nicotinamide with accuracy and precision comparable with that obtained by HPLC or GC techniques. Thus, the developed method could be applied alternatively to both techniques in laboratory control of pharmaceuticals containing nicotinamide (as* Vitamin PP*).

## 4. Conclusions

In conclusion, the research study followed in this work confirmed the applicability of silica gel 60 RP18WF_254_ plates and also methanol-water in volume composition 3 : 7 for the quantitative determination of nicotinamide (as* Vitamin PP*) in selected pharmaceutical formulations (e.g., tablets and injection solution) by the use of HPTLC-densitometric technique in reversed-phase system. Validation of proposed method showed that the developed RPHPTLC-densitometric method allowed to determine the nicotinamide content (as* Vitamin PP*) in tablets and injection solution in the presence of its related substances, for example, N,N-diethylnicotinamide, N-methylnicotinamide, and nicotinic acid with accuracy and precision comparable to that obtained using HPLC or GC methods, respectively. Therefore, the proposed method can be successfully applied in routine laboratory control of pharmaceutical formulations containing nicotinamide.

## Figures and Tables

**Table 1:** Statistical analysis of nicotinamide content in its commercial drugs using the proposed RPHPTLC-densitometric method.

Parameter	Pharmaceutical products		
	Nicotinamide tablets *(Vitamin PP)* *(Product 1) *	Nicotinamide tablets *(Vitamin PP)* *(Product 2) *	Nicotinamide injection solution *(Vitamin PP)* *(Product 3) *
Number of determinations (*n*)	6	6	6
Label claimed of nicotinamide (mg)	200	200	100
Mean amount of nicotinamide (mg)	198.4	198.6	99.4
Minimum amount of nicotinamide (mg)	195.9	195.8	97.5
Maximum amount of nicotinamide (mg)	202.2	201.2	101.2
Variation (*s* ^2^)	4.80	3.10	1.53
Standard deviation (SD)	2.2	1.8	1.2
Coefficient of variation (CV, %)	1.11	0.91	1.21
The 95% confidence interval of arithmetic mean	*μ* = 198.4 ± 2.2	*μ* = 198.6 ± 1.8	*μ* = 99.4 ± 1.2
Percentage amount of nicotinamide found (%) in relation to declared value	99.2%	99.3%	99.4%
